# Aberrant disgust response and immune reactivity in cocaine-dependent men might uncover deranged serotoninergic activity

**DOI:** 10.3389/fnmol.2014.00007

**Published:** 2014-02-11

**Authors:** Carmelo M. Vicario

**Affiliations:** ^1^School of Psychology, The University of QueenslandBrisbane, QLD, Australia; ^2^School of Psychology, The University of BangorBangor, UK

**Keywords:** cocaine dependence, disgust, immune reactivity, serotonin, sympathetic nervous system

In a recent issue of Biological Psychiatry, Ersche et al. (2014) published an interesting study on disgust sensitivity in Cocaine-dependent men. The authors of this research have explored the immunomodulatory effects of cocaine in disgust sensitivity, which is considered an important psychological mechanism of protection against the exposure to pathogens. Therefore, these researchers used neutral and disgust-evoking photographs depicting food and nonfood images while response accuracy, latency, and skin conductance were recorded. The skin conductance response was investigated as measure of the activity of the sympathetic system. Moreover, saliva samples were collected before and after exposure to neutral and disgusting images, in order to examine the effect played by these visual stimuli to the immune system reactivity of this clinical population. In particular, it was examined the levels of cytokine interleukin-6 (IL-6), which is a key regulator of inflammatory processes in response to acute infection (Gabay and Kushner, 1999).

The results are intriguing as they show aberrant skin conductivity and increased secretion of the salivary cytokine interleukin-6 relative to the exposure to disgusting images in the cocaine dependent individuals relative to a group of healthy control subjects. This was taken as evidence of a hypersensitivity to disgusting stimuli in cocaine-dependent individuals. More specifically, the authors interpreted their finding as possible evidence of conditioned responses to non-ingestive sources of infection.

The increased sympathetic and immune responses documented in this clinical population in association to a hypersensitivity to stimuli conveying a risk of infection might be explained in relation to a deranged activity of serotoninergic circuits. This suggestion stems from at least three different arguments.

First, it is well known that cocaine blocks reuptake of serotonin and other neurotransmitters into presynaptic neurons by binding to the neuronal membrane transporters for this monoamine (Ritz et al., 1990). It has been also shown that cocaine suppresses serotonin synthesis, leading to decreased tissue levels of serotonin and its metabolite, the 5-hydroxyindoleacetic acid (5-HIAA) (Baumann et al., 1993). Moreover, human postmortem studies have shown decreased serotonin transporter binding sites, quantified with the radioligand [123I] 2-carbomethoxy-3-(4-iodophenyl) tropane ([123I]β-CIT), in drug users with concurrent opiate use (Little et al., 1998). Finally, there is evidence (e.g., Fan et al., 1994, 1995; Breitinger et al., 2001) documenting a direct inhibition of serotonin type 3 (5HT3) receptors by cocaine.

Second, serotonin has been recently discussed in relation to its important role in the processing of disgust and aversiveness. For example, Limebeer et al. (2004) have shown that depletion of forebrain serotonin (5-HT) by 5,7-dihydrox-ytryptamine (5,7-DHT) lesions prevents induced conditioned disgust reactions such as “gaping,” the predominant conditioned rejection reaction (Parker, 2003) to “taste related” aversive stimulation. Wright et al. (2010) have also shown that learning to avoid odors associated with the malaise caused by ingesting toxins is mediated by serotonin (see also Vicario, 2013a,b, for recent discussions about this argument). Moreover, there is evidence of altered serotoninergic activity in Anorexia Nervosa (AN) (e.g., Jean et al., 2012), a psychiatric disorder characterized by a marked disgust sensitivity for food (Vicario and Candidi, 2011; Vicario and Crescentini, 2012; Vicario, 2013c).

Last but not the least, the work of Rubio-Godoy et al. (2006) argues that 5-HT might be the link between disgust and immunity. In fact, 5-HT not only plays a central role in both the induction of the emetic reflex and the learned aversion, but it is also a signal used by immune cells to modulate both innate and acquired immunity. For example, Janeway et al. (2005) have shown that 5-HT is a potent pro-inflammatory signal and upregulates phagocytosis in peritoneal macrophages; moreover, 5-HT might be used as a neurotransmitter by the immune system. This is suggested by the finding of dendritic cells delivering this compound to T cells across the immunological synapse in a manner similar to that which occurs between neurons (Bird, 2005). It is interesting to note that a deranged serotoninergic activity might also explain the aberrant skin conductivity in the cocaine-dependent individuals examined by Ersche et al. (2014). In fact, there is evidence that serotonin influences sympathetic activity (see Ducy and Karsenty, 2010; Zimmerman et al., 2012). For example, it was recently shown that serotonin might increase the sympathetic preganglionic neurons current-evoked firing frequency in neonatal mice (Zimmerman et al., 2012).

All these investigations converge in the suggestion that a deranged serotoninergic activity might represent the key factor linking disgust hypersensitivity, aberrant immune response and increased sympathetic tone in cocaine-dependent men.
